# IMPROVE 2025: The 3rd International Meeting on Pathway‐Related Obesity: Vision & Evidence

**DOI:** 10.1111/cob.70109

**Published:** 2026-09-10

**Authors:** Jesús Argente, Erica L. T. van den Akker, Metin Cetiner, Karine Clément, Elizabeth Forsythe, Alastair Garfield, Peter Kühnen, Gabriel Ángel Martos‐Moreno, David Meeker, Hermann L. Müller, Hanneke M. van Santen, Julia von Schnurbein, Bérénice Segrestin, Tomohiro Tanaka, Manuel Tena‐Sempere, Martin Wabitsch, Stefanie Zorn, I. Sadaf Farooqi

**Affiliations:** ^1^ Department of Paediatrics and Paediatric Endocrinology Hospital Infantil Universitario Niño Jesús, Research Institute La Princesa Madrid Spain; ^2^ CIBER Fisiopatología de la Obesidad y Nutrición (CIBEROBN) Instituto de Salud Carlos III Madrid Spain; ^3^ Department of Paediatrics Universidad Autónoma de Madrid Madrid Spain; ^4^ IMDEA Food Institute Madrid Spain; ^5^ Department of Paediatric Endocrinology Erasmus MC Children's Hospital Rotterdam the Netherlands; ^6^ Department of Paediatrics II University Hospital Essen, University of Duisburg‐Essen Essen Germany; ^7^ INSERM, Nutrition and Obesities: Systemic Approaches, NutriOmics, Research Unit Sorbonne Université Paris France; ^8^ Assistance Publique‐Hôpitaux de Paris, Nutrition Department Pitié‐Salpêtrière Hospital Paris France; ^9^ Guy's and St Thomas' Hospitals, Great Ormond Street Hospital London UK; ^10^ Rhythm Pharmaceuticals, Inc. Boston Massachusetts USA; ^11^ Department of Paediatric Endocrinology and Diabetology Charité Universitätsmedizin Berlin, Corporate Member of Freie Universität Berlin and Humboldt‐Universität Berlin Berlin Germany; ^12^ German Centre for Child and Adolescent Health (DZKJ), Partner Site Berlin Berlin Germany; ^13^ Department of Paediatrics and Paediatric Hematology/Oncology University Children's Hospital, Carl von Ossietzky Universität, Klinikum Oldenburg AöR Oldenburg Germany; ^14^ Department of Paediatric Endocrinology Wilhelmina Children's Hospital, University Medical Center Utrecht the Netherlands; ^15^ Princess Máxima Centre for Paediatric Oncology Utrecht the Netherlands; ^16^ Division of Paediatric Endocrinology and Diabetes, Department of Paediatrics and Adolescent Medicine University Medical Centre Ulm Ulm Germany; ^17^ Department of Endocrinology, Diabetes and Nutrition Hôpital Lyon Sud Hospices Civils de Lyon Pierre‐Bénite France; ^18^ CarMeN Laboratory, INSERM, INRA, INSA Lyon Université Claude Bernard Lyon 1 Hospices Civils de Lyon Pierre‐Bénite France; ^19^ Department of Gastroenterology and Metabolism Nagoya City University Graduate School of Medical Sciences Nagoya Aichi Japan; ^20^ Department of Cellular Biology, Physiology and Immunology, and Instituto Maimonides de Investigacion Biomedica de Córdoba (IMIBIC) University of Córdoba Córdoba Spain; ^21^ German Centre for Child and Adolescent Health (DZKJ), Partner Site Ulm Ulm Germany; ^22^ Institute of Metabolic Science and NIHR Cambridge Biomedical Research Centre University of Cambridge Cambridge UK

**Keywords:** acquired hypothalamic obesity, Bardet–Biedl syndrome, hyperphagia, MC4R pathway, monogenic disease

## Abstract

An international cohort of 161 clinicians and researchers from 19 countries attended the 3rd International Meeting on Pathway‐Related Obesity: Vision & Evidence (IMPROVE) in Prague, Czech Republic, on 2–4 July 2025. The aims of the meeting were to advance understanding of hyperphagia and obesity caused by defects in the melanocortin‐4 receptor (MC4R) pathway and to support peer‐to‐peer sharing of clinical experience and emerging evidence. The programme comprised four sessions covering hyperphagia, monogenic MC4R pathway diseases, Bardet–Biedl syndrome and acquired hypothalamic obesity. Discussions focused on defining and measuring hyperphagia, updates in genetic diagnostics and natural history, real‐world and trial data on targeted therapies and practical multidisciplinary models of care with earlier intervention. Patient and caregiver quality of life remained central throughout. These Proceedings summarise the key updates, discussions and initiatives generated at IMPROVE 2025.

## 1 Introduction

The 3rd International Meeting on Pathway‐Related Obesity: Vision & Evidence (IMPROVE) took place in Prague, Czech Republic, on July 2–4, 2025, bringing together 18 faculty members and 143 delegates from 19 countries. IMPROVE 2025 focused on advancing the understanding of hyperphagia and rare melanocortin‐4 receptor (MC4R) pathway‐related diseases, including monogenic diseases, Bardet–Biedl syndrome (BBS) and acquired hypothalamic obesity (aHO). Through keynote lectures, expert presentations and interactive workshops, participants explored emerging genetic insights, real‐world evidence and multidisciplinary approaches to diagnosis and treatment. Workshops facilitated cross‐specialty dialogue on care models for young patients, comorbidity management and treatment strategies for rare MC4R pathway‐related diseases. IMPROVE 2025 underscored the importance of collaborative research and integrated care in addressing the complexities of pathway‐related obesity. In this report, we consolidate key data and insights and summarise focal discussions and initiatives that characterised the meeting.

## 2 KEYNOTE Lecture: Hypothalamic Control of Body Weight: Understanding Obesity and Its Treatment

*Professor Manuel Tena‐Sempere, University of Córdoba and CIBER* ‘Fisiopatologia de la obesidad y nutricion’ *(CIBEROBN), Instituto de Salud Carlos III, Madrid, Spain*.

Between 1990 and 2021, overweight and obesity increased substantially in every world region, with children and adolescents being particularly affected [1]. Increases in obesity incidence are expected to continue, with forecasts suggesting that by 2050, 360 million individuals 5–14 years of age will be living with obesity [1]. Compelling genetic, clinical and experimental evidence endorses the important contribution of brain circuits to obesity [2]. In particular, the hypothalamus contains major regulatory circuits for bodyweight homeostasis, the deregulation of which can lead to obesity [2]. The hypothalamus integrates peripheral, environmental and neural sensory inputs to influence a range of physiological functions and behaviours such as hunger, satiety, thermoregulation and energy homeostasis [3], pubertal development [4] and levels of reproductive hormone.

In the hypothalamus, energy balance is coordinated by two interlocking pathways: an anorexigenic system that promotes satiety and an orexigenic system that stimulates hunger. Within the arcuate nucleus (ARC), proopiomelanocortin (POMC)‐expressing neurons process the POMC precursor to produce alpha‐melanocyte‐stimulating hormone (α‐MSH), which delivers an anorexigenic signal through melanocortin receptors. In parallel, neuropeptide Y (NPY) and agouti‐related peptide (AgRP) neurons provide an orexigenic drive that increases appetite. The interplay between these neuronal populations underpins hypothalamic control of hunger and satiety [2].

Hypothalamic AMP‐activated protein kinase (AMPK) has a major role as a canonical modulator of energy balance through integration of peripheral signals, such as hormones and metabolites, within neuronal networks [5]. AMPK is implicated in the regulation of feeding, thermogenesis in brown adipose tissue and browning of white adipose tissue [5]. Evidence also suggests that hypothalamic AMPK signalling could be a target for anti‐obesity drug development [5]. A study has shown that the body weight of mice with obesity can be reduced by intravenous injection of small extracellular vesicles delivering a plasmid encoding a dominant negative mutant of AMPKα1‐targeted to steroidogenic factor 1 neurons at the ventromedial nucleus of the hypothalamus. This underscores the potential for AMPK‐targeted therapies to manipulate body weight and reduce obesity [6]. Glucagon‐like peptide‐1 receptor agonists (GLP‐1 RAs), such as liraglutide, also exert part of their anti‐obesity effects via interactions with AMPK [5]. Dr. Tena‐Sempere presented unpublished data from his laboratory obtained in a mouse model of diet‐induced obesity that illustrated how liraglutide decreased body weight, food intake, adiposity, insulin resistance and leptin levels while also increasing glucose tolerance.

Many genes are involved in the hypothalamic circuits that regulate energy homeostasis and food intake, such as the leptin receptor (*LEPR*), the prohormone convertase 1/3 (*PCSK1*), *POMC, SH2B1, NCOA1* and *MC4R* genes. Variants in these genes and others, can lead to hunger/hyperphagia, reduced energy expenditure and the development of obesity [2]. Leptin, produced in adipose tissue, has a key role in modulating appetite and energy balance by acting on neurons in the hypothalamus that express LEPR, such as POMC neurons [2]. POMC peptides in these neurons are processed by PCSK1 to produce α‐MSH, which binds to MC4R in target neurons to initiate downstream activity that influences energy expenditure, food intake and hunger/satiety [2].

Food intake can have a profound effect on pubertal development, with animal models showing that under‐ and over‐feeding (malnutrition or obesity) can disrupt pubertal timing [M. Tena‐Sempere, unpublished data and V. Sobrino, unpublished data]. Kisspeptin‐expressing neurons in the ARC are crucial for the metabolic control of puberty [7]. These neurons contribute to the initiation of puberty by stimulating gonadotropin‐releasing hormone (GnRH) neurons and also integrate metabolic signals to ensure puberty occurs when sufficient energy reserves are available [7].

In addition to the anorectic effects of the neuropeptide α‐MSH from POMC neurons, α‐MSH also has a role in the metabolic regulation of puberty through effects on the kisspeptin/GnRH neuronal signalling pathway that regulates the permissive effects of leptin on pubertal maturation [4]. Furthermore, POMC and its derivative α‐MSH influence the hypothalamic–pituitary–gonadal axis through regulation of GnRH release, which impacts luteinizing hormone levels essential for controlling the reproductive cycle [M. Tena‐Sempere, unpublished data]. Melanocortin‐3 receptors (MC3R) are also important in linking caloric sufficiency signalled through POMC‐expressing neurons to the control of growth and reproduction [8].

Rodent models of polycystic ovary syndrome (PCOS) have shown that an excess of androgens is linked to overweight/obesity [9]. However, hypothalamic proteomics in androgenic obesity models report limited numbers of differentially expressed factors in animals with obesity [V. M. Serrano unpublished data]. Treatments investigated in animal models of PCOS concluded that a GLP‐1 RA/oestrogen conjugate [10] has superior efficacy to correct obesity and metabolic complications over either of the hormones alone, tirzepatide, (GLP‐1 RA/glucose‐dependent insulinotropic polypeptide) and metformin (diabetes treatment) [9].

Taken together, the studies highlight the role of the hypothalamus as a major integratory hub pivotal to metabolic control, feeding, thermogenesis, body weight control and onset of puberty and reveal the potential for therapeutic targets.

## 3 Diagnosis and Treatment of Hyperphagia

*Professor Erica L. T. van den Akker, Erasmus MC Children's Hospital, Rotterdam, the Netherlands*.

Hyperphagia, a pathological, insatiable hunger leading to excessive preoccupation with food, increased food intake and early‐onset obesity, is a key symptom of rare MC4R pathway diseases and has a profound effect on the quality of life of patients (e.g., aggressive behaviour, sleep problems, low self‐esteem, stigmatisation) and their caregivers. In fact, hyperphagia may be viewed as a disease itself, separate from obesity.

Measurement of hyperphagia is currently challenging given that physicians, patients and caregivers may have limited awareness of symptoms and patients may not always recognise or disclose their hyperphagia due to associated stigma. In addition, the medical community has no standard definition for hyperphagia [11] and without such a standardisation, development of accurate measurement tools has proven complex. This is problematic as the ability to measure food/caloric consumption and behaviours accurately is critical in quantifying whether excess or insufficient energy intake is occurring, with consequent decisions around necessity and direction of diagnostics and the effectiveness of interventions.

Further complicating measurement is the lack of validated tools to assess for hyperphagia in patients with monogenic or syndromic diseases [12]. In a study by Yuan et al. [13] a semiquantitative food frequency questionnaire was shown to be reasonably correlated/validated in assessing nutrient intakes in comparison with a standard 7‐day dietary record (food diary) [13]. However, food diaries do not cover all aspects of eating behaviour such as satiety, emotional eating, restrictive eating or binge eating. In 2025, Heymsfield et al. proposed a set of simple questions used to screen patients for hyperphagia in a general clinical setting, which addressed satiety, preoccupation with food and abnormal food‐related behaviours. Questions like these could provide the foundation for a dedicated hyperphagia questionnaire [12].

A validated questionnaire originally designed to specifically assess hyperphagic behaviour in patients with Prader–Willi syndrome (PWS), the Hyperphagia Questionnaire for Clinical Trials (HQ‐CT), has now been evaluated in a wider and more general population (individuals 5–26 years of age) [14]. The study found that overall, the HQ‐CT captures significant differences in hyperphagia‐associated behaviours between controls and individuals with PWS. However, the study also reported some limitations of the tool, including self‐reporting bias, insufficient information around body mass index (BMI) and lack of information about food security measures [14]. In support of the value of the HQ‐CT, a further study by Zorn et al. [15] reported that the questionnaire was a simple and useful tool in clinical practice to assess the quality and severity of hyperphagia in patients living with obesity due to monogenic and syndromic disease who had a preoccupation with food and demonstrated food‐seeking behaviour [15]. Additional tools used in practice include the Eating Behaviour Questionnaire [16], containing targeted questions to score on three themes: restrictive eating, emotional eating and external eating; however, it was not developed for hyperphagia specifically and its use in screening for hyperphagia needs to be assessed. Finally, a straightforward Visual Analogue Scale (VAS), scoring hunger at momentary points in time, could be useful especially for tracking progress in individual patients, but not all patients are able to complete a VAS themselves and it is not suitable for comparing across patients, limiting its use as a subjective tool.

**FIGURE 1:**
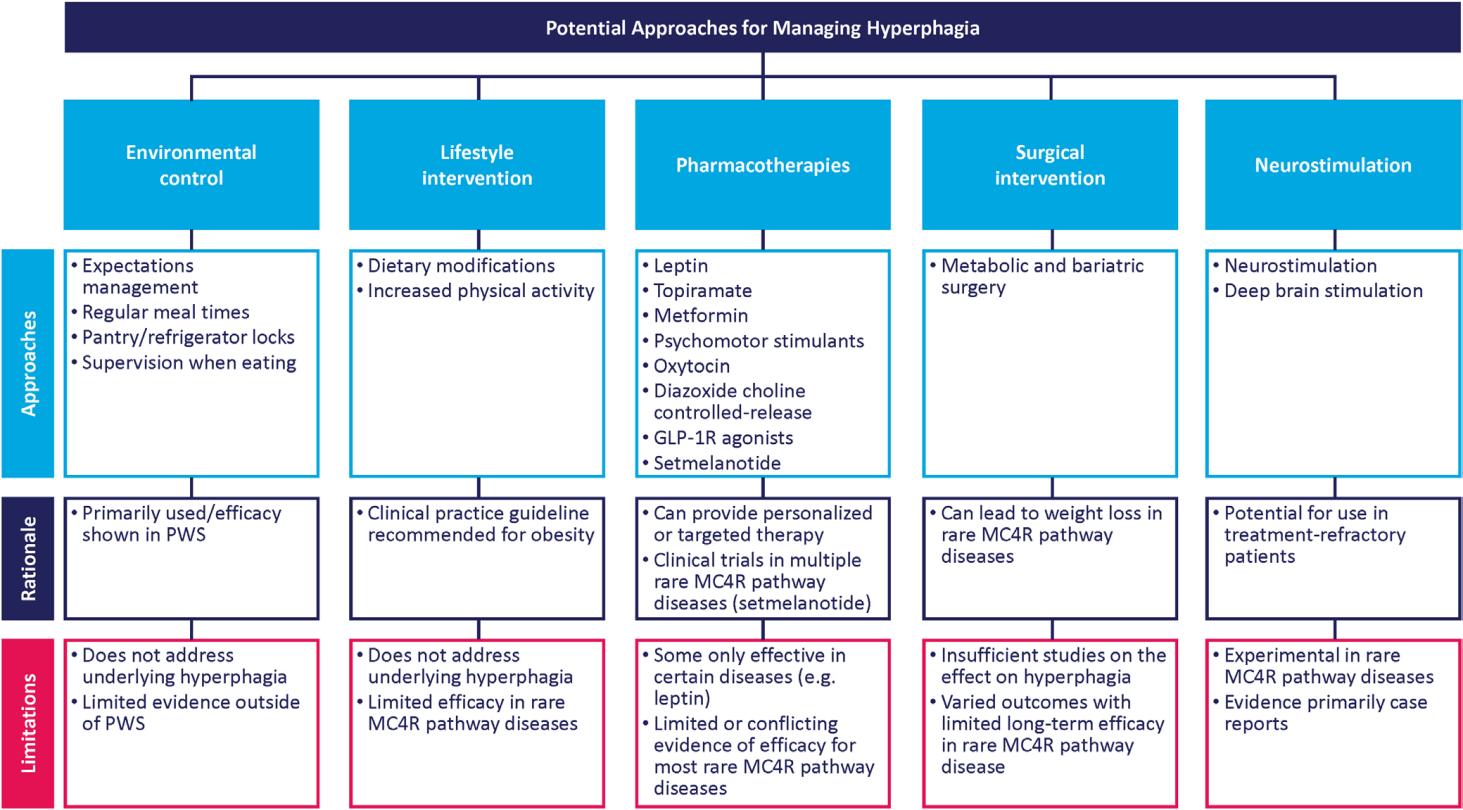
Potential approaches for the management of hyperphagia [11]. GLP‐1 R, glucagon‐like peptide‐1 receptor; MC4R, melanocortin‐4 receptor; PWS, Prader–Willi syndrome. Figure reproduced and adapted from Argente et al. [11], distributed under the terms of the Creative Commons Attribution (CC‐BY) licence.

In addition to the lack of validated tools for identifying and measuring hyperphagia, currently no standard treatment guidelines exist for the management of hyperphagia. A recent paper by Argente et al. [11] has attempted to address the lack of standardisation by proposing a treatment algorithm for managing hyperphagia based on five key pillars: environmental control, lifestyle intervention, pharmacotherapies, surgical intervention in care and neurostimulation in experimental phase (see Figure 1). The algorithm provides an overview of the management approaches available, alongside the rationale for and limitations of each pillar. For environmental control, it's important to provide regular mealtimes—no exceptions—and prohibit access to food. Lifestyle intervention includes dietary modifications and increased physical exercise, while surgical interventions focus on metabolic and bariatric surgery and neurostimulation involves deep‐brain stimulation. A wide range of pharmacotherapies are also available, allowing for personalised or targeted therapy and include common GLP‐1 RAs and the targeted MC4R agonist setmelanotide [11].

## 4 Monogenic Diseases

### 4.1 Regulators of Leptin–Melanocortin Signalling

*Professor I. Sadaf Farooqi, University of Cambridge, Cambridge, UK*.

As outlined, energy homeostasis is regulated by the leptin–melanocortin pathway [17]. POMC deficiency and PCSK1 deficiency lead to obesity, lack of adrenocorticotropic hormone and multiple neuroendocrine abnormalities [18]. Research has shown that loss‐of‐function variants in the *MC4R* gene can result in hyperphagia, severe obesity, increased linear growth and lean mass (together with higher bone mineral density) and disproportionate hyperinsulinemia [19]. Upstream defects, including congenital leptin or LEPR deficiency, similarly disrupt signalling to POMC neurons and cause hyperphagia with early‐onset obesity [2, 17].

Pleiotropic obesity syndrome genes also disrupt melanocortin circuits, resulting in diseases such as BBS, pseudohypoparathyroidism or PWS, in which learning difficulties and organ‐specific abnormalities predominate [20]. Furthermore, studies have identified that hypothalamic melanocortin circuits modulate multiple components of eating behaviour, such as preference for high amounts of fat or a reduced preference for high sucrose‐containing foods [21].

Recognising a possible leptin–melanocortin pathway disease in children with early‐onset obesity is challenging. Obesity is often the dominant presenting problem, so the genetic aetiology can be missed and many patients are not referred for testing despite co‐occurring developmental delay or neurobehavioural features. Identifying specific genetic variants that contribute to an individual's hyperphagia and obesity can potentially lead to more targeted treatments and management strategies and increase the effectiveness of therapies employed. A candidate gene for targeted therapy is *GPR101*, which is expressed in leptin‐responsive GABAergic neurons distinct from POMC and AgRP populations [22]. Functional studies have demonstrated that GPR101 regulates leptin‐receptor–mediated signalling directly, boosting leptin‐induced STAT3 activation up to fivefold, thereby enhancing leptin sensitivity in GABAergic neurons [23, 24], [I. S. Farooqi, unpublished data]. Analysis of exome data from the Genetics of Obesity Study (GOOS) cohort and UK Biobank identified rare *GPR101* variants (S296G, V423M, D486G) among patients with severe obesity. Of note, seven of eight (88%) carriers of the S296G variant had obesity compared with 24% of non‐carriers, suggesting that impaired GPR101 function reduces leptin signalling and contributes to obesity [I. S. Farooqi, unpublished data]. These findings implicate GPR101 as a novel regulator of leptin–melanocortin signalling, linking leptin resistance through GABA neurons to severe early‐onset obesity.

In summary, dysregulation of central appetite control, centred on the leptin–melanocortin pathway, is a primary driver of severe early‐onset obesity. Genetic insights, from loss‐of‐function *MC4R* variants to emerging targets such as GPR101 in leptin‐responsive GABAergic neurons, map the circuits that shape these diseases and identify novel therapeutic targets. Clinical guidelines recommend genetic testing as part of the diagnostic evaluation for severe early‐onset obesity, typically before 5 years of age, to guide management and trial eligibility, as well as to reduce stigma by recognising the biological basis of disease.

### 4.2 New Genes Involved in the MC4R Pathway

*Dr Gabriel Ángel Martos‐Moreno, Hospital Infantil Universitario Niño Jesús, Research Institute La Princesa; CIBER* ‘Fisiopatología de la obesidad y nutrición *(CIBEROBN), Instituto de Salud Carlos III; Universidad Autónoma de Madrid, Madrid, Spain*.

Hypothalamic melanocortin neurons sit at the core of energy homeostasis. Beyond *LEP*, *LEPR*, *POMC*, *PCSK1* and *MC4R*, several other genes implicated in human obesity converge on this axis. At the level of POMC regulation, *SH2B1* [25], *NCOA1* [26] and *PHIP* [27] modulate leptin‐receptor signalling and *POMC* transcription and pathogenic variants in these genes associated with severe early‐onset obesity have been described. On the receptor side, MC3R and MC4R functions are shaped by *MRAP2* [28], *ADCY3* [29] and *GNAS* [30], which influence cAMP signalling and receptor trafficking, including localisation to primary cilia. Developmental control adds a further layer: the transcription factor TBX3 directs the terminal specification and postnatal maintenance of melanocortin neuron identity [31] and loss of TBX3 in hypothalamic (particularly POMC) neurons causes weight gain and metabolic disturbances [32]. In parallel, members of the *SEMA3* family, with *PLXNA* and *NRP* co‐receptors, guide the development of hypothalamic projections and variants in these genes have been linked to cases of human obesity [33]. Primary cilia proteins, including components of the BBSome and related ciliopathy genes, also affect receptor positioning and melanocortin output [34].

These converging mechanisms identify discrete, genotype‐defined populations with MC4R pathway disruption. Setmelanotide, a selective MC4R agonist, has been shown to effectively reduce hunger/hyperphagia and weight in subpopulations with POMC, PCSK1 or LEPR deficiency and with BBS [35, 36, 37].

DAYBREAK is a two‐stage, multi‐country, Phase 2 study of setmelanotide in patients with hyperphagia and obesity who carry confirmed MC4R‐pathway variants (*PHIP*, *PLXNA* [1, 2, 3, 4], *SEMA3[A–G]*, *SIM1*, *MAGEL2*, *TBX3* and *RPGRIP1L*). Adults entered with BMI ≥ 40 kg/m^2^ and children at ≥ 97th percentile [38, 39]. Stage 1 was an open‐label run‐in where the primary endpoint was the proportion of responders within each genotype achieving a ≥ 5% reduction in BMI from baseline. Responders entered Stage 2, a randomised, double‐blind, placebo‐controlled withdrawal period, with Stage 2 efficacy assessed by change in BMI from baseline to the end of Stage 2 (*n* = 39). A higher proportion of patients in the setmelanotide arm (*n* = 27, 84.4%), compared to placebo (*n* = 5, 29.4%), achieved or maintained 5% BMI reduction from study baseline to the end of Stage 2 and the mean percent BMI change in the continuous setmelanotide arm from baseline to the end of Stage 2 was **−**12.4%. Patients with the *SEMA3E* variant showed the largest reductions in BMI, while those with *SEMA3* (A, D, F, G) variants experienced a more variable degree of weight loss compared to placebo. Patients with *PLXNA* (1–4) and *SIM1* variants demonstrated weight‐loss responses, which were heterogeneous and were better explained by the degree of functional impairment observed in post hoc functional analyses than by American College of Medical Genetics and Genomics (ACMG) variant classification alone. In contrast, individuals with *PHIP* variants exhibited a more consistent and sustained weight loss response. Setmelanotide was well tolerated with no new safety concerns across the entire study [39].

Further studies may elucidate whether the genetic variants explored in DAYBREAK contribute to a loss of function in the MC4R pathway or identify other patient‐specific factors that can impact response to setmelanotide treatment [40].

### 4.3 Care for Patients With Rare Genetic Obesity

#### 4.3.1 Findings From a First Multicentre Collaboration Project

*Dr Stefanie Zorn, University Medical Centre Ulm, Ulm, Germany*.

In up to 3% of children with severe obesity, the disease is caused by monogenic biallelic variants affecting the MC4R pathway [41, 42, 43]. A study by Zorn et al. [44] investigated the natural course of height, weight and BMI from birth up to 5 years of age in a pooled European cohort of patients with monogenic MC4R pathway disease. The study also aimed to establish cut‐off values for specific anthropometric parameters with the highest sensitivity to facilitate the identification of patients for future genetic screening. Patients included in the study were diagnosed with biallelic (likely) pathogenic variants in *LEP, LEPR*, *POMC*, *PCSK1* and *MC4R* genes or monoallelic (likely) pathogenic variants in the *MC4R* gene [44].

Forming the largest European cohort, a total of 147 patients with monogenic MC4R pathway disease were included. Individuals with biallelic *LEP*, *LEPR* and *MC4R* variants had similar early childhood BMI development, characterised by a steep increase in BMI in the first year of life, followed by a plateau in BMI values. Patients with biallelic *POMC* variants showed a rapid increase in BMI over the course of the first 2 years of life followed by a gradual flattening of the BMI curve. Patients with monoallelic *MC4R* variants showed a steady BMI increase during early childhood and had a significantly lower BMI than patients with biallelic variants from the age of 6 months onwards. While patients with biallelic variants in *LEP* and *LEPR* showed normal body length development, patients with biallelic or monoallelic *MC4R* variants or biallelic *POMC* variants showed accelerated growth by exceeding the 97th percentile of the World Health Organization growth reference from 1 year of age onwards. In terms of establishing cut‐off values for genetic testing, the best time point to discriminate between patients with biallelic variants affecting genes in the MC4R pathway and control patients with severe obesity without a known genetic cause (*n* = 113) was at 2 years of age with an optimal BMI cut‐off value of 24 kg/m^2^.

Incorporation of these new data into clinical practice will enable earlier diagnosis and access to targeted interventions for patients with monogenic MC4R pathway diseases, potentially leading to more specific recommendations for genetic screening.

#### 4.3.2 Launch of a Registry for Future Research

*Dr Julia von Schnurbein, University Medical Centre Ulm, Ulm, Germany*.

Patients living with obesity due to monogenic variants, such as those affecting the MC4R pathway, are rare; therefore, most centres see only a few patients. Fragmented experience limits understanding of natural history, comorbidities, modifying factors such as parental BMI and real‐world treatment response. The International Registry for Genetic Obesity (iGO) will address this limitation by establishing a large pan‐European and international database of patients living with obesity that has a confirmed genetic cause or is strongly suspected because of early‐onset obesity. It will capture a standardised baseline profile and yearly follow‐ups to allow detailed characterisation over time and to support nested studies in subsets of the cohort as new research questions arise. The registry allows for capturing a total of 300 data elements, of which 100 will belong to a core dataset, organised into basic and episode forms. Basic forms capture informed consent, patient status, demographics, genetic characteristics, birth history, obesity history, family history, prior obesity treatments and sample availability. Episode forms capture new information at each visit such as anthropometry, body composition, physical examination findings, comorbidities across systems (metabolic, psychological, orthopaedic, neuromuscular, neurological, endocrine, cardiovascular and other illnesses), laboratory parameters, therapies and patient‐reported outcomes. The registry will use the Open Source Registry System for Rare Diseases infrastructure with high data security and automatic pseudonymisation. Data ownership will remain with patients and their treating physicians and participating clinicians will govern the registry through an elected board. The iGO will enable efficient multicentre collaboration, accelerating discovery, informing trials and improving care for people living with obesity due to monogenic variants.

## 5 Bardet–Biedl Syndrome

### 5.1 Treatment in BBS: Paediatric Outcomes and Metabolic Impact—From Sequence to Significance: Variant Interpretation in Genetic Obesity Panels

*Dr Elizabeth Forsythe, Guy's and St Thomas' Hospitals, Great Ormond Street Hospital, London, UK*.

BBS is a rare autosomal recessive ciliopathy linked to variants in at least 26 genes and 4 modifier genes [45]. Primary cilia are required for correct localisation and functioning of the leptin receptor on the cell membrane and ciliary dysfunction can impair leptin‐receptor signalling, which in turn may disrupt MC4R signalling, ultimately driving hyperphagia and early‐onset obesity [45, 46]. The phenotype is pleiotropic, may be detectable in utero and evolves with age [45], with clinical features including retinal dystrophy, hyperphagia, early‐onset obesity, post‐axial polydactyly, renal dysfunction, learning difficulties and hypogonadism [45, 46].

#### 5.1.1 Setmelanotide Improves Weight and Quality of Life in Children With MC4R Pathway Diseases Aged 2–5 Years

Setmelanotide has been shown to reduce hunger and weight effectively in patients ≥ 6 years of age with rare MC4R pathway diseases such as POMC, PCSK1 or LEPR deficiency or with BBS [35, 36]. Setmelanotide is now also approved by the FDA and EMEA for children ≥ 2 years of age in POMC, PCSK1, LEPR deficiency and BBS [37].

VENTURE was a Phase 3, open‐label, multicentre study at six sites in the USA, UK, Spain and Australia. It enrolled children 2–5 years of age with hyperphagia and obesity due to biallelic *POMC* (including *PCSK1*) or *LEPR* variants or genetically confirmed BBS (*N* = 12) [37]. Setmelanotide was administered once daily for 52 weeks, starting at 0.5 mg with 0.5‐mg increases every 2 weeks to a weight‐based maximum of 0.5–2.0 mg. The co‐primary endpoints at Week 52 were the percentage of patients reaching a ≥ 0.2‐point decrease in BMI z‐score and mean percent change in BMI. Additional endpoints assessed safety, hunger, weight‐related outcomes and caregiver burden [37].

In the overall cohort, 83% (*n* = 10/12) of patients achieved a ≥ 0.2‐point decrease in BMI *z*‐score at Week 52, with a mean change of −3.4 (standard deviation [SD] 2.5). The mean percentage change in BMI was −18% (*n* = 11). The mean change from baseline in the percent of the 95th percentile for BMI (%BMI95) and waist circumference was −32.5 and −7.7 cm, respectively and the Zarit Burden Interview score improved by −13.2 points, indicating a reduced perceived burden of caregiving. Caregivers reported less hunger versus baseline in 91% of children (*n* = 10/11). No new safety signals were observed [37].

When looking at the patients with BBS (*n* = 5), 80% (*n* = 4/5) achieved a ≥ 0.2‐point decrease in BMI z‐score at Week 52, with a mean change of −1.3 (SD 1.2). The mean percentage change in BMI was −10% (*n* = 5). The mean change from baseline in %BMI95, waist circumference and the Zarit Burden Interview was −14.5, −2.0 and −13.2 cm, respectively. Caregiver‐reported hunger improved versus baseline in 80% (*n* = 4/5) of patients [37].

Taken together, 1 year of setmelanotide was associated with reductions in weight‐related measures and hunger in children 2–5 years of age with POMC/LEPR deficiency or BBS, alongside no new safety concerns, supporting early, mechanism‐based treatment in this population [37].

#### 5.1.2 Impact of Setmelanotide on Metabolic Syndrome Risk in Children With BBS

Patients with BBS are at increased risk for cardiometabolic complications. In a Phase 3 trial in patients with BBS ≥ 6 years of age, setmelanotide showed significant hunger and weight reductions and improved metabolic parameters [36]. These outcomes prompted the hypothesis that patients with BBS treated with setmelanotide could also experience a decreased risk of metabolic syndrome (MetS), along with associated reduced risks of cardiovascular disease and type 2 diabetes. To estimate the risk and severity of MetS in patients with BBS, investigators used the MetS‐Z‐BMI score algorithm that has been created using data from the US National Health and Nutrition Examination Survey and combines age‐, sex‐ and race‐/ethnicity‐specific coefficients with BMI z‐score in children (BMI in adults), HDL cholesterol, triglycerides, fasting glucose and systolic blood pressure [47, 48]. Since no European algorithm is validated, patients of European origin were mapped to the closest US race/ethnicity category. This study drew on paediatric participants from the Phase 3 trial of setmelanotide in patients with BBS (NCT03746522) [36] who had complete data to calculate MetS‐Z‐BMI at baseline and Week 52 and identifiable age, sex and race/ethnicity for coefficient assignment (*n* = 13) [49]. Patients were also stratified by a prespecified 1‐year weight response (achievers defined as a ≥ 0.3‐point reduction in BMI *z*‐score).

At baseline, the mean (SD) MetS‐Z‐BMI score was 1.04 (0.48) and scores were higher in patients with *BBS1* than with *BBS10* variants (1.23 [0.30] versus 0.74 [0.47]; *p* = 0.043). After 52 weeks of setmelanotide, the mean (SD) change in MetS‐Z‐BMI was −0.13 (0.61) and in BMI z‐score was −0.35 (0.37). Improvements concentrated in clinical responders: weight‐threshold achievers (*n* = 6) had a mean (SD) change in MetS‐Z‐BMI of −0.52 (0.54) versus +0.20 (0.47) in non‐achievers (*n* = 7), representing a between‐group difference of 0.72; *p* = 0.025. Given the small sample and exploratory nature, the *p* value should be interpreted cautiously. Haqq et al. published a full analysis of this study which also included nine adult patients in addition to the 13 paediatric patients presented here [49].

Overall, 1 year of setmelanotide was associated with reductions in a composite metabolic‐risk score, pointing to a possible reduction in future cardiovascular and type 2 diabetes risk in paediatric patients with BBS. Improvements were greatest in patients who met the prespecified weight threshold, underscoring the utility of MetS‐Z‐BMI as an additional measure of treatment benefit alongside hyperphagia and weight. A limitation of the MetS‐Z‐BMI algorithm is that it can give higher scores to patients whose metabolic parameters sit at the upper end of normal, so results should be interpreted with that in mind.

#### 5.1.3 Variant Interpretation of Genetic Obesity Panels

General obesity and obesity from underlying genetic causes are distinct diseases; although they may involve overlapping neurobiological pathways [50], they are not the same. Obesity due to rare genetic variants is a highly penetrant disease that clinicians need to distinguish from general obesity. Therefore, a genetic diagnosis matters. It can guide day‐to‐day care, point to targeted treatment, clarify prognosis and trigger cascade testing and family‐planning conversations when relatives may be at risk.

Variant interpretation should follow the ACMG standards and guidelines [51]. Only Class 4 (likely pathogenic) and Class 5 (predicted to be pathogenic) findings are used to direct medical care. Class 3 variants remain uncertain and require follow‐up, re‐examination or additional studies. Results typically fall into one of three outcomes: a confirmed genetic diagnosis that fits the inheritance pattern; no disease‐causing variant found; or a variant of uncertain significance that requires more evidence before acting or disregarding.

Obesity panel choice should be driven by the clinical question and local availability. Key aspects to compare include the scope of coverage, which variant classes are reported and the laboratory's policy on variants of uncertain significance, whether variants for syndromic diseases such as PWS and BBS are included and accreditation and expected turnaround time. For example, the UK NHS early severe obesity panel covers 35 entities across 32 genes and three chromosomal regions, reports Class 4 and 5 variants, does not report carrier status and the turnaround is > 3 months. The Rhythm Pharmaceuticals Rare Obesity Advanced Diagnosis (ROAD) genetic testing programme spans 89 entities across 88 genes and one chromosomal region (June 2026), excludes PWS, can report Class 3–5 variants, reports carrier status and typically returns results in about 3–4 weeks.

Real‐world cases have shown how this works in practice. When red flags for a rare genetic cause were present—early onset, severe obesity with hyperphagia, neurodevelopmental features and family histories suggesting a particular mode of inheritance—clinicians selected an appropriate genetic obesity panel to confirm diagnosis. Diagnostic results were managed by confirming segregation, arranging counselling for relatives, issuing ‘To Whom It May Concern’ letters where needed and discussing reproductive options. When panels did not reveal a diagnosis, the message was to keep an open mind about genetics and, where relevant, revisit testing as new evidence or broader panels become available.

Overall, panel choice should be deliberate, results interpreted within ACMG criteria, action taken only on Class 4 or 5 findings in the correct inheritance context and all results paired with clear counselling, given that test outcomes direct management, follow‐up and family screening.

### 5.2 French BBS Real‐World Evidence Data

*Professor Karine Clément, INSERM, Sorbonne Université and Pitié‐Salpêtrière Hospital, Paris, France*.

Rare genetic MC4R pathway diseases need coordinated, personalised care with early genetic testing, structured transition from paediatrics to adult services and timely access to targeted treatment. France has generated real‐world, post‐authorisation follow‐up data from an early access programme for setmelanotide [52]. Patients are managed in a specialised centre with a multidisciplinary approach at Pitié‐Salpêtrière Hospital in Paris. Between January 2022 and December 2024, 17 people were followed: 12 who started setmelanotide in care and five who continued treatment from clinical trials. Diagnoses included BBS in 11 patients and biallelic variants in *LEPR* (*n* = 4) or *POMC* (*n* = 2). Baseline characteristics for starters were (mean values): age 30.8 years, weight 130.6 kg, BMI 48.0 kg/m^2^ and waist circumference 129.2 cm; and for continuers were (mean values): age 34.6 years, weight 131.2 kg, BMI 45.5 kg/m^2^ and waist circumference 134.0 cm. Starters were close to their historical maximum weight (mean 96.7% of maximum), while continuers were at 71.1%, reflecting earlier loss during setmelanotide trials. Mean follow‐up across the dataset was 14.4 months.

Weight trajectories demonstrated the impact of setmelanotide on weight loss. In those starting treatment, the percentage of maximum weight fell by 20% at 12 months, with inter‐patient variability in the pace and extent of loss. In those continuing therapy after a trial, earlier reductions were maintained, with individual trajectories dispersed but stable across 50–108 months of ongoing drug treatment.

Beyond weight, hunger scores improved in patients who initiated setmelanotide and importantly food cravings fell by 41% at 12 months. Quality of life also improved as assessed by the Impact of Weight on Quality of Life‐Lite (IWQOL‐Lite). The global IWQOL‐Lite score increased from baseline to 12 months and domain plots showed gains across mobility, self‐esteem, social life, work life and sexual life. The safety profile of setmelanotide in the real world was similar to that of the Phase 3 clinical trials, with hyperpigmentation experienced by all patients. The presentation underscored the value of multidisciplinary support to sustain adherence, called for better characterisation of changes in eating behaviour across dimensions and proposed to introduce therapy in patients < 6 years of age, within a multiprofessional pathway.

Taken together, these real‐world data show that starting setmelanotide is associated with clinically relevant 1‐year reductions in weight measures and hunger, while continued therapy preserves prior weight loss over many years. These observations support sustained multidisciplinary follow‐up for people with BBS or biallelic *LEPR* or *POMC* variants.

### 5.3 New Clinical Findings and Patient Management

*Dr Metin Cetiner, University Hospital Essen, University of Duisburg‐Essen, Essen, Germany*.

As real‐world experience grows, emerging data suggest that the benefits of setmelanotide in patients with BBS extend beyond hunger and body weight, with additional signals across metabolic health and organ function [53].

A prospective, single‐centre cohort study from University Children's Hospital of Essen (June–December 2023) evaluated patients > 6 years of age with genetically confirmed BBS, hyperphagia and/or obesity, starting setmelanotide, with ultrasound‐based liver assessment (attenuation imaging [ATI] and shear‐wave elastography), bioimpedance and a hyperphagia questionnaire (Impact of Hyperphagia and Symptoms of Hyperphagia; maximum total score 50) alongside standard clinical measures. After 6 months (*n* = 26), BMI z‐score fell by −0.5 (95% confidence interval [CI] −0.6 to −0.3), hyperphagia scores decreased by −12.3 (95% CI −15.5 to −9.0), total body fat fell by −3.1% (95% CI −5.7 to −0.5), estimated glomerular filtration rate increased by up to 10.1 mL/min/1.73 m^2^ (95% CI 4.3–15.9) and liver attenuation improved (ATI −0.14 dB/cm/MHz; 95% CI −0.17 to −0.11). Notably, > 80% of patients showed resolution of metabolic dysfunction‐associated steatotic liver disease (MASLD) or stabilisation at grade S1 (mild) and MASLD improvement correlated with liver size rather than weight change, consistent with effects independent of BMI reduction [53]. This organ‐level focus builds on earlier characterisation work from the same centre, which documented frequent kidney and liver involvement in BBS and highlighted the utility of quantitative ultrasound parameters for detecting steatosis and fibrosis markers in routine care [54].

Adverse events (AEs) were most often reported early in treatment, with skin pigmentation changes and gastrointestinal symptoms among the common AEs at initiation. Clear expectation setting and practical support for patients are necessary, with dose‐management approaches, such as slower up‐titration or temporary down‐titration, to manage tolerability rather than stop treatment. These points align with the approved product information, which lists hyperpigmentation, injection‐site reactions, nausea, headache and diarrhoea among the most frequent reactions and notes sexual adverse reactions including spontaneous erections [55, 56].

A complementary real‐world survey from the University Children's Hospital of Essen captured 6‐month treatment experience with a specialist nurse support programme among 35 respondents (10 paediatric patients, 13 adult patients, 12 caregivers) who began setmelanotide between June and December 2023. Before treatment, insatiable hunger and obesity were the most burdensome symptoms for children and caregivers and vision loss and obesity predominated in adults. After ~6 months of treatment, ≥ 92% across survey groups reported feeling less hungry, feeling satiated after meals and experiencing weight loss or weight stabilisation; the mean BMI z‐score (± SD) at baseline was 3.12 ± 0.89 with a mean change of −0.47 ± 0.37 at 6 months. Mobility, mood and behaviour also improved. The nurse service, designed to provide early, practical support with education, injection training and problem‐solving, was rated ‘excellent’ by all respondents, enabled most patients or caregivers to administer injections independently and supported high adherence, with no discontinuations during the observation window [55].

Taken together, these real‐world data show consistent reductions in hyperphagia and age‐appropriate weight measures, alongside improvements in liver parameters and kidney function in selected patients, highlighting the value of multidisciplinary support and personalised titration. In practice, assessments should look beyond hunger and weight at metabolic and organ measures when judging benefit over time [53, 55].

## 6 Acquired Hypothalamic Obesity

### 6.1 aHO Review

*Professor Hanneke M. van Santen, Wilhelmina Children's Hospital and Princess Máxima Centre for Paediatric Oncology, Utrecht, the Netherlands*.

Disruption of hypothalamic signalling can impair MC4R pathway function, leading to dysregulation of energy balance. This disruption may arise from genetic causes, including syndromic and non‐syndromic rare MC4R pathway diseases, such as BBS, PWS or rapid‐onset obesity with hypoventilation, hypothalamic, autonomic dysregulation, neuroendocrine tumour (ROHHADNET) syndrome [57].

Structural damage to the hypothalamic region may occur due to tumours (e.g., craniopharyngioma, germ cell tumours, hypothalamic glioma), surgical or tumour‐related treatment, inflammation or trauma [57]. This damage disrupts hypothalamic control of hormonal, metabolic and autonomic processes, affecting heart rate, blood pressure, temperature and circadian rhythm. These disturbances often cause hyperphagia and reduced energy expenditure, resulting in accelerated and sustained weight gain that is a characteristic of acquired hypothalamic obesity (aHO) [58, 59].

This presentation focused on aHO, which most often develops following hypothalamic injury due to lesions such as craniopharyngioma or glioma, after which patients may develop pituitary insufficiency, behavioural problems, hyperphagia, morbid obesity, hypothermia and sleep problems [58, 59, 60]. aHO is characterised by hyperphagia with accelerated and sustained weight gain, but is often accompanied by neurobehavioural and psychiatric abnormalities (such as attention deficit and reduced impulse control), sleep problems, decreased energy expenditure, pituitary dysfunction and temperature dysregulation [57, 61]. All these factors can be used as part of the diagnostic criteria and all need to be addressed as part of individualised management [60].

Recognising aHO can be difficult but recently developed diagnostic criteria for signs and symptoms of hypothalamic dysfunction, the underlying driver of aHO, may aid in early recognition and diagnosis, in the reporting and understanding of its aetiology and in predicting its course and management [62]. Treatment for hypothalamic dysfunction requires an experienced multidisciplinary team, with centralisation of care in dedicated centres to ensure improvements in BMI outcomes, specifically a reduction in aHO [63]. Similarly to general obesity, aHO management involves lifestyle changes such as improved diet and engaging in physical activity [64]. Pharmaceutical treatment should ideally be initiated within 3 months of surgery [65], as the steepest rise in BMI typically occurs during this early postoperative period [65]. Current pharmacological options include metformin, diazoxide, GLP‐1 RAs and dexamphetamines [66, 67]; however, robust evidence supporting their efficacy in clinical trials is limited.

Setmelanotide has also been investigated in patients with aHO in the Phase 3 TRANSCEND study [68, 69]. The double‐blinded, 52‐week trial enrolled 120 participants and is the largest and longest placebo‐controlled trial to evaluate a targeted therapy for patients with aHO. Patients treated with setmelanotide (*n* = 81) had a mean BMI reduction of −16.5% from baseline compared with +3.3% BMI change for patients on placebo (*n* = 39) at 52 weeks (*p* < 0.0001), equating to a −19.8% placebo‐adjusted difference in BMI reduction. Furthermore, adult patients ≥ 18 years of age treated with setmelanotide had a −19.2% placebo‐adjusted BMI reduction at 52 weeks and patients < 18 years of age had a −20.2% placebo‐adjusted reduction [68]. These findings suggest that setmelanotide represents an additional targeted pharmacological option for patients with aHO.

**FIGURE 2:**
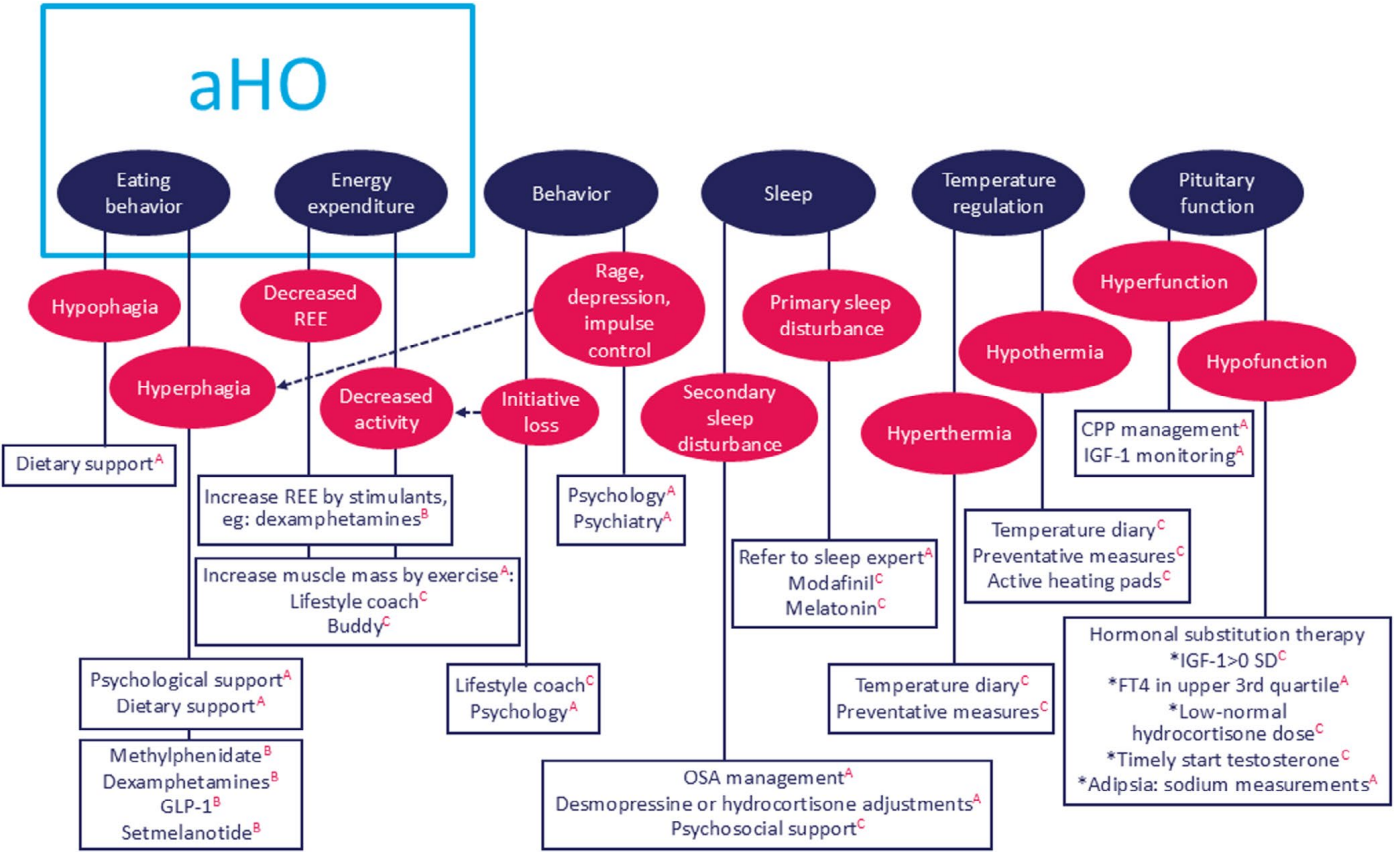
Algorithm for the management of acquired hypothalamic dysfunction [70]. Suggested interventions are categorised as follows: ^A^existing guidelines, ^B^clinical trial or case series data, ^C^expert opinion. aHO, acquired hypothalamic obesity; CPP, central precocious puberty; FT4, free thyroxine; GLP‐1, glucagon‐like peptide‐1; IGF‐1, insulin‐like growth factor 1; OSA, obstructive sleep apnea; REE, resting energy expenditure; SD, standard deviation. Figure reproduced and adapted from van Santen et al. [70], distributed under the terms of the Creative Commons Attribution (CC‐BY) licence.

Because of new data and a deeper understanding, an updated treatment algorithm for hypothalamic dysfunction, including aHO, has been proposed. It focuses on six domains—eating behaviour, energy expenditure, behaviour, sleep, temperature regulation and pituitary function—with early, individualised intervention in specialist centres guided by the diagnostic score (see Figure 2) [70].

### 6.2 Key Findings From Claims Databases

#### 6.2.1 The European Perspective

*Professor Hermann L. Müller, University Children's Hospital, Carl von Ossietzky Universität, Klinikum Oldenburg AöR, Oldenburg, Germany*.

In Germany, several analyses have been performed to better understand the epidemiology and healthcare burden of aHO. Using national claims data, a database algorithm was developed that allows for the standardised identification of patients with aHO due to tumour/treatment‐related HO (TTR‐aHO) [71]. The algorithm analysed a representative claims database of 5.42 million individuals covering the years 2010–2020. Two clear incidence peaks of aHO were observed: one among children and young adults 10–24 years of age and another among adults 40–44 years of age. The overall incidence rate of TTR‐aHO was 6.8 per million people, with a higher proportion of cases occurring in females. The tumours most frequently associated with aHO were benign sellar and suprasellar lesions, including craniopharyngiomas, pituitary neoplasms and other non‐specific endocrine tumours [71].

To better understand treatment pathways for patients living with TTR‐aHO, a further analysis of German claims data was carried out during the 2 years following an index surgical treatment [72]. Claims from 37 patients with TTR‐aHO were analysed on a quarterly basis over 2 years, with inpatient, outpatient and prescription data considered in the analysis. In the 2‐year follow‐up period, patients with TTR‐aHO were hospitalised 3.67 times on average, with 37% of hospitalisations in the first year and 31% in the second year. Over the follow‐up period, on average, patients saw a general practitioner 12.27 times and various specialists 20.49 times. In addition, the need for complex neuroendocrine therapy developed quickly, with most patients having 2 or 3 neuroendocrine prescriptions in any given quarter [72].

A third study in Germany, using the same claims database of 5.42 million patients, evaluated healthcare resource use and costs for patients with TTR‐aHO across hospitalisations, outpatient visits, visits per specialist group and outpatient prescription medications [73]. Compared with non‐HO obesity, those with TTR‐aHO had a substantially higher healthcare utilisation, including approximately six times more hospitalisations, nearly twice as many outpatient visits and over two times more prescribed medications in the 2 years following tumour surgery or radiotherapy. Moreover, costs for patients with TTR‐aHO were higher compared with patients with non‐aHO, with excess costs of ~€19 900 per patient in the first year and ~€10 700 in the second, driven primarily by inpatient costs. Cost‐intensive hormone replacement therapies, such as somatropin, were a leading contributor to increased prescription costs in the second year [73].

**FIGURE 3:**
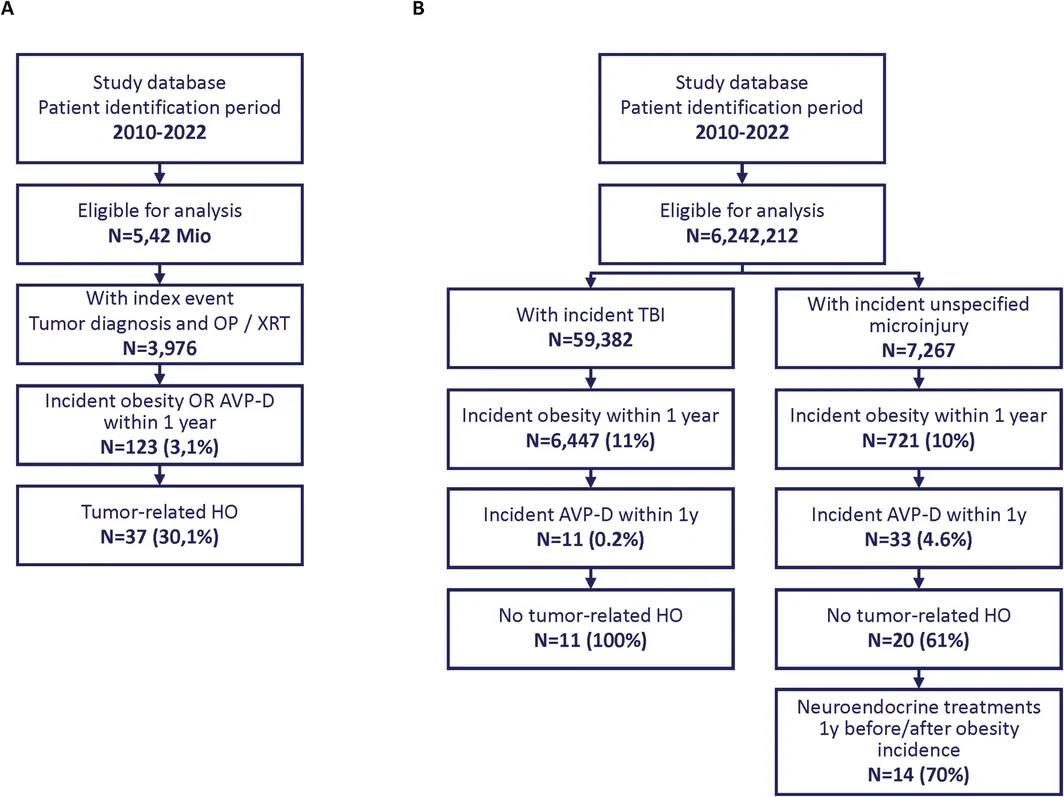
Epidemiological results of German claims data analyses (collected between 2010 and 2020) for (A) TTR‐aHO [71] and (B) aHO in patients with TBI and UM (data collected between 2010 and 2022) [74, 75]. aHO, acquired hypothalamic obesity; AVP‐D, arginine vasopressin deficiency; OP, surgery; TBI, traumatic brain injury; TTR‐aHO, tumour/treatment‐related aHO; UM, unspecified microinjury; XRT, irradiation. Figure A and B reproduced from Witte et al. [74] and Müller et al. [75], distributed under the terms of the Creative Commons Attribution (CC‐BY) licence.

In a fourth analysis of the German claims database [74], the role of traumatic brain injury (TBI) and hypothalamic microinjury in aHO was further analysed (Figure 3). Among more than 6.2 million insured individuals, 59 382 incident TBIs were identified, 11% of whom developed obesity within 1 year. Only a small subset (11 patients) developed confirmed aHO with documented arginine vasopressin deficiency (AVP‐D) or pituitary dysfunction, none of which were tumour‐related. Among 7267 individuals with unspecified hypothalamic microinjury, 10% developed obesity within 1 year and 4.6% exhibited incident AVP‐D. After excluding tumour‐related cases, 20 patients remained. Following the diagnosis of incident obesity, most patients in both cohorts received at least two neuroendocrine therapies in addition to desmopressin, most commonly including hydrocortisone and levothyroxine sodium.

The management of patients with TTR‐aHO requires frequent inpatient and outpatient visits for tumour follow‐up and management of incident comorbidities and most patients with TTR‐aHO require intense polytherapy. Comprehensive characterisation of patients with aHO will be key not only for developing effective treatment strategies and improving patient outcomes, but also for understanding the impacts on healthcare resource use and economic burden associated with the disease.

#### 6.2.2 The Japanese Perspective

*Dr Tomohiro Tanaka, Nagoya City University Graduate School of Medical Sciences, Nagoya, Aichi, Japan*.

In Japan, a single‐centre pilot study examined the estimated epidemiology of aHO [T. Tanaka, unpublished data]. Patients were included if they had been diagnosed with pituitary hormone deficiency (*N* = 67); mean age at diagnosis was 31 years, 40% were undergoing surgery, 15% were being treated with radiotherapy and 27% with chemotherapy. Prevalence of obesity, defined in Japan as BMI ≥ 25 kg/m^2^, was 34% overall but 46% in those patients with a tumour and a hypothalamic lesion, which were both higher than the average 25% among the general population in Japan. Individual case reports of patients having surgery for craniopharyngioma or pituitary neuroendocrine tumour demonstrated an accelerated and sustained change in weight trajectory following the surgery [T. Tanaka, unpublished data].

To understand the epidemiology of aHO more precisely, a nationwide hospital database in Japan evaluated the proportion of patients ≥ 18 years of age with underlying diseases known to affect the hypothalamus who experienced accelerated weight gain following index hospitalisation. Three criteria were used to validate aHO: weight gain (≥ 15% increase in BMI) after index hospitalisation, presence of additional diagnoses of hormone deficiency or associated medication and sustained obesity (BMI ≥ 25 kg/m^2^) after 12 months. Among patients with craniopharyngioma, 12.3% met all three validation criteria for aHO, with most cases showing accelerated weight gain within 12 months. Similar patterns of weight gain were also observed in patients with either pituitary or germ cell tumours. While aHO was proportionally most frequent in adult patients with craniopharyngioma, the majority of total aHO cases in adults were attributable to other, more prevalent brain tumours [76].

These data underline the importance of comprehensive patient profiling to guide targeted treatment strategies and improve long‐term outcomes in aHO. Further research is required to explore additional causes, including TBI, stroke and other neurological insults.

### 6.3 Real‐World Evidence Data for aHO in France

*Dr Bérénice Segrestin, Hôpital Lyon Sud, Hospices Civils de Lyon, CarMeN Laboratory, INSERM, Pierre‐Bénite, France*.

In contrast to general obesity, which often responds to conventional lifestyle and pharmacological interventions, hyperphagia and weight gain in aHO are typically challenging to manage due to underlying hypothalamic dysfunction [58, 59]. In France, setmelanotide is available to patients with aHO under a pre‐marketing early‐access authorisation. A real‐world analysis evaluated outcomes in paediatric and adult patients with aHO (*n* = 25) who received ≥ 3 months of setmelanotide treatment. The real‐world population included mostly patients with craniopharyngioma, but other etiologies, such as astrocytoma, inflammation and septo‐optic dysplasia were also present [77], [B. Segrestin, unpublished data]. Setmelanotide was initiated at a mean age of 12.7 years in paediatric patients and 31.5 years in adult patients. Approximately 50% of the adult patients, but none of the paediatric patients, had received or were continuing treatment with a GLP‐1 RA.

In paediatric patients with aHO, BMI *z*‐scores decreased from baseline at all timepoints analysed, with reductions of up to 0.4 points (10.6%) at 9 months of treatment with setmelanotide. Similarly, in adults with aHO, BMI decreased from baseline at all timepoints analysed, with up to a 23% reduction at 9 months. Hunger scores also improved over the 9‐month period although the study was not designed to capture hunger scores accurately. In paediatric patients with aHO due to anatomic defects (*n* = 4), BMI z‐scores decreased by 0.2 at 3 months and by 0.4 at 6 months. The single adult patient with aHO due to Rathke's cleft cyst experienced a decrease in BMI of 14.8% after 6 months of setmelanotide treatment. Overall, no new safety signals were observed, with most AEs reported being injection‐site reactions, hyperpigmentation and nausea.

The real‐world data show a relevant clinical benefit of setmelanotide treatment on hunger and BMI in paediatric and adult patients 6–42 years of age with aHO, consistent with the findings of Phase 2 and 3 trials of setmelanotide in aHO [68, 69, 78].

## 7 Workshop Highlights

### 7.1 Workshop 1: Care for Patients With Rare MC4R Pathway Disease

*Professor Erica L. T. van den Akker, Erasmus MC Children's Hospital, Rotterdam, the Netherlands; Professor Martin Wabitsch*, University Medical Centre Ulm and German Centre for Child and Adolescent Health (DZKJ), Partner Site Ulm, Ulm, Germany.

This workshop aimed to identify and discuss on a regional level the approaches to management of MC4R pathway disruption and provide a forum for peer‐to‐peer sharing of experiences.

The delegates agreed that patients with rare MC4R pathway diseases need a structured chronic care pathway owing to complications developing early in life, impaired quality of life and mental health, psychosocial stress caused by stigmatisation and impaired body image and self‐esteem. Further agreement centred on the need for individualised treatment provided by a multidisciplinary team and a planned transition pathway to ensure continuity between paediatric and adult care.

The delegates also agreed that resources needed for a structured care programme include training materials for healthcare professionals (HCPs), families and patients, a case manager to provide a point of contact and reassurance, patient support groups and continued screening for comorbidities. Furthermore, barriers to supplying a structured care programme were identified that include the lack of a dedicated nurse specialist, insufficient education programmes for HCPs, failure to recognise obesity as a medical specialty and the need to improve non‐stigmatising communication.

### 7.2 Workshop 2: Multidisciplinary Care and Treatment Perspectives for Patients Aged Under 6 Years

*Professor Peter Kühnen, Charité Universitätsmedizin Berlin and German Centre for Child and Adolescent Health, Berlin, Germany; Professor Jesús Argente, Hospital Infantil Universitario Niño Jesús; Universidad Autónoma de Madrid; CIBER* ‘Fisiopatologia de la obesidad y nutricion’ *(CIBEROBN), Instituto de Salud Carlos III and IMDEA Food Institute, Madrid, Spain*.

This workshop aimed to examine multidisciplinary collaboration in the care of patients with rare MC4R pathway diseases < 6 years of age and explore perspectives on optimal management strategies.

The delegates agreed that the onset of clinical manifestations of BBS is variable, making diagnosis challenging. Furthermore, a multidisciplinary approach to diagnosis was considered key and early treatment deemed crucial for improving quality of life and preventing the development of longer‐term complications. Discussion also focused on the rarity of LEPR deficiency as a disease, emphasising the need for collaboration and sharing experiences.

Further discussions centred on the need for a team that should be comprised of a physician specialised in obesity, a nephrologist, an ophthalmologist, a gastroenterologist, a psychologist, a paediatric endocrinologist, a dietician/nutritionist, a physiotherapist, a paediatric neurologist, a social worker, a specialised nurse, a dermatologist, a paediatric cardiologist and a paediatric pulmonologist. However, it was also acknowledged that the composition of a multidisciplinary team will differ per rare MC4R pathway disease.

Consensus was reached on the individual nature of every patient's needs and the conviction that treatment effect may differ in each patient, owing to variation in genetics, differences in dosing of treatments, contrasting abilities in terms of physical activity and, pertinently, whether hyperphagia has been addressed. Treatment success needs to be critically and regularly reviewed by the multidisciplinary team to enable the adaptation or modification of a treatment strategy with the aim of improving the health status and quality of life of the patient.

### 7.3 Workshop 3: Management of Comorbidities and Comedications in Patients With aHO

*Professor Hanneke M. van Santen, Wilhelmina Children's Hospital and Princess Máxima Centre for Paediatric Oncology, Utrecht, the Netherlands; Professor Hermann L. Müller, University Children's Hospital, Carl von Ossietzky Universität, Klinikum Oldenburg AöR, Oldenburg, Germany*.

aHO is characterised by accelerated and sustained change in weight trajectory leading to morbid obesity. This is often accompanied by a range of comorbidities arising both from underlying hypothalamic dysfunction and from aHO itself. This includes multiple endocrine abnormalities (e.g., hypopituitarism), with or without adipsia, neurobehavioural impairments, circadian rhythm disruption, reduced energy expenditure, low core body temperature and increased risk of cardiovascular and metabolic disorders [70]. These factors may coexist and interact, with potential additive or synergistic effects; for example, aHO may contribute to cardiovascular risk independently of and in addition to, obesity‐related mechanisms [79]. This workshop aimed to review and share practice on the management of comorbidities that can occur in patients with aHO and appraise comedication approaches to management.

Consensus was reached on the use of growth hormone (GH) as a treatment option for supporting muscle strength and bone health, which are often affected in hypothalamic dysfunction. Treatment with GH must always be evaluated carefully depending on the type and aggressiveness of the tumour and the presence of residual unresectable tumour. In general, GH can be started 3 months after tumour removal of a benign tumour such as craniopharyngioma or pituitary adenoma and 1 year after achieving complete remission after treatment for a malignant tumour such as germinoma. GH is not to be used for hyperphagia management or weight reduction. Participants also agreed on initiating setmelanotide 3 months after surgery, if available and it was recommended to reduce the dose of setmelanotide temporarily if AEs were becoming unmanageable and to up‐titrate again once they were controlled.

Delegates acknowledged that recognising aHO and hypothalamic syndrome may be difficult due to its rarity and variety of symptoms. Diagnostic criteria for signs and symptoms of hypothalamic dysfunction may aid in early recognition and diagnosis [70] and in the reporting and understanding of its aetiology. It may also help in predicting the disease course and planning management strategies. Weight management can be challenging due to treatment‐related side effects and permissive family attitudes towards food intake during recovery from tumour treatment. Better education of patients and their families regarding post‐surgery implications was also seen as crucial, with many patients and families not understanding that hyperphagia and aHO can be a consequence of surgery, indicating the need for improved pre‐operative counselling.

## 8 Clinical and Scientific Update

### 8.1 MC4R Agonism: Beyond POMC

*Alastair Garfield and David Meeker, Rhythm Pharmaceuticals Inc., Boston, Massachusetts, USA*.

Setmelanotide is currently approved for the treatment of obesity and the control of hunger in adults and children ≥ 2 years with POMC, PCSK1, LEPR deficiency and BBS. Setmelanotide was also recently approved by the FDA and EMEA in adults and children ≥ 4 years with aHO.

Setmelanotide has proven efficacy in the treatment of individuals with diverse MC4R pathway diseases [80, 81]. However, residual signalling through AgRP neurons may lead to a reduced treatment effect in patients with BBS or LEPR deficiency, resulting in unchecked hyperphagia and continued reduced energy expenditure, suggesting the need for different (potentially higher) dosing regimens and/or earlier intervention in these patient populations. This may reflect underlying pathophysiology; in BBS and LEPR deficiency, defects upstream of MC4R reduce activation of POMC neurons, while inhibitory signalling via AgRP neurons is preserved. This ongoing AgRP‐mediated antagonism of MC4R may attenuate the response to MC4R agonists.

New populations for MC4R pathway therapy are being explored across an ongoing clinical programme, including the Phase 2 DAYBREAK (gene variants strongly associated with the MC4R pathway), the Phase 3 EMANATE (heterozygous variants in MC4R pathway‐associated genes), the Phase 3 TRANSCEND (aHO) and an open‐label PWS study.

In DAYBREAK, setmelanotide was associated with variable weight‐related responses in patients with different genetic variants in the genes *PCSK1*, *plexin A* (*PLXNA*), *semaphorin‐3* (*SEMA‐3*) and *single‐minded homologue 1* (*SIM1*), suggesting that further studies may elucidate whether the genetic variants explored in this trial contribute to a loss of function in the MC4R pathway or can identify other patient‐specific factors that can modulate response to setmelanotide treatment [38, 39].

In TRANSCEND, the primary endpoint was met, with a −19.8% placebo‐adjusted reduction in BMI at 52 weeks (at therapeutic dose) of setmelanotide treatment. Setmelanotide also significantly reduced the weekly average of the maximal daily hunger score in patients with aHO ≥ 12 years of age compared with placebo, as well as significantly reducing the impact of hyperphagia on both paediatric patients and their caregivers (including quality of life and physical functioning) [68].

In an initial Phase 2 proof‐of‐concept study in PWS (completed in 2016), no overall difference versus placebo was seen at the lower dose ranges, although some patients receiving higher doses (2.5 mg) showed notable weight decreases [82]. A working hypothesis is that imprinting and gene‐dosage effects involving *MAGEL2* in the PWS critical chromosomal region may have influenced treatment response in these patients with PWS, consistent with preclinical data showing hypersensitivity to setmelanotide in *MAGEL2*‐deficient mice [83].

Unpublished data show that individual patients with *MAGEL2* variants have an observed response to setmelanotide, with 30% of patients with the deficiency achieving a ≥ 5% reduction in BMI [Rhythm Pharmaceuticals Inc. unpublished data]. The clinical response to setmelanotide in a patient with MAGEL2 deficiency enrolled in the Phase 2 DAYBREAK trial further supports the PWS treatment rationale, with persistent weight loss, reduced waist circumference and reduced hunger scores observed [Rhythm Pharmaceuticals Inc. unpublished data]. To further expand on these encouraging data, setmelanotide is currently being investigated in an open‐label Phase 2 study of patients with PWS to evaluate the impact of dosages of ≥ 1–5 mg over a 6‐month period on weight‐related changes and hyperphagia scores [84]. The Prader–Willi‐like population is another disease that may benefit from targeted MC4R agonist therapy owing to high prevalence rates of hyperphagia and obesity [85].

Restoring normal physiology through targeting the MC4R pathway may be key to countering obesity through reductions in hyperphagia and increases in energy expenditure.

## 9 Summary and Meeting Close

IMPROVE 2025 accomplished its aim to advance the understanding of rare MC4R pathway diseases across monogenic diseases, BBS and aHO. The faculty linked new biology to day‐to‐day practice, with clear implications for diagnosis, treatment choice and service design.

The meeting established clear priorities: incorporate genetic testing into early pathways and use to guide care and family screening; treat the mechanism and address hyperphagia directly, since reducing hyperphagia improves daily functioning and quality of life in the short‐term, as well as reducing obesity; support adherence and manage AEs to sustain benefit of targeted treatment; provide care that is multidisciplinary, with clear transition between paediatric and adult services, better education for patients and families and non‐stigmatising communication in the clinic setting.

New data and initiatives are helping to accelerate progress in managing rare MC4R pathway diseases, in particular BBS and LEPR deficiency. Real‐world evidence in BBS and emerging aHO datasets show meaningful reductions in hyperphagia and improvements in weight‐related and patient‐reported outcomes. Recent FDA and EMEA approvals for setmelanotide in aHO and further controlled trials are further expanding this evidence base.

In summary, IMPROVE 2025 strengthened the understanding and approach to managing rare MC4R pathway diseases. The meeting underlined the need for greater collaboration across multiple disciplines and how an individualised, patient‐centred holistic approach to care using multifaceted treatment strategies is crucial for addressing the complexities of these diseases and ultimately improving outcomes for patients.

Taken together, the discussions at IMPROVE 2025 set a clear agenda: diagnose earlier, target biology, ease hyperphagia, capture outcomes in a structured way and collaborate across specialties and centres. This patient‐centred, multidisciplinary‐team–based approach offers the clearest route to better long‐term outcomes for patients with MC4R pathway diseases.

## Funding

This work was supported by Rhythm Pharmaceuticals Inc.

## Ethics Statement

Ethics approval and informed consent were not required for this article because it is a congress proceedings publication that summarises and discusses data previously presented at a scientific meeting and does not report any new research involving human participants.

## Conflicts of Interest

All presenters received speaker fees and travel reimbursement from Rhythm Pharmaceuticals Inc. for speaking at the meeting.

## Data Availability

Data sharing is not applicable to this article as no new data were created or analysed in this article.

## References

[1] GBD 2021 Collaborators , “Global, Regional, and National Prevalence of Child and Adolescent Overweight and Obesity, 1990–2021, With Forecasts to 2050: A Forecasting Study for the Global Burden of Disease Study 2021,” Lancet 405, no. 10481 (2025): 785–812. 40049185 10.1016/S0140-6736(25)00397-6 PMC11920006

[2] J. Argente , I. S. Farooqi , J. A. Chowen , et al., “Hypothalamic Obesity: From Basic Mechanisms to Clinical Perspectives,” Lancet Diabetes and Endocrinology 13, no. 1 (2025): 57–68. 39547253 10.1016/S2213-8587(24)00283-3

[3] H. Fong , J. Zheng , and D. Kurrasch , “The Structural and Functional Complexity of the Integrative Hypothalamus,” Science 382, no. 6669 (2023): 388–394. 37883552 10.1126/science.adh8488

[4] M. Manfredi‐Lozano , J. Roa , F. Ruiz‐Pino , et al., “Defining a Novel Leptin‐Melanocortin‐Kisspeptin Pathway Involved in the Metabolic Control of Puberty,” Molecular Metabolism 5, no. 10 (2016): 844–857. 27688998 10.1016/j.molmet.2016.08.003 PMC5034608

[5] M. Lopez , R. Nogueiras , M. Tena‐Sempere , and C. Dieguez , “Hypothalamic AMPK: A Canonical Regulator of Whole‐Body Energy Balance,” Nature Reviews. Endocrinology 12, no. 7 (2016): 421–432. 10.1038/nrendo.2016.67 27199291

[6] E. Milbank , N. R. V. Dragano , I. González‐García , et al., “Small Extracellular Vesicle‐Mediated Targeting of Hypothalamic AMPKα1 Corrects Obesity Through BAT Activation,” Nature Metabolism 3, no. 10 (2021): 1415–1431. 10.1038/s42255-021-00467-8 34675439

[7] M. A. Sanchez‐Garrido and M. Tena‐Sempere , “Metabolic Control of Puberty: Roles of Leptin and Kisspeptins,” Hormones and Behavior 64, no. 2 (2013): 187–194. 23998663 10.1016/j.yhbeh.2013.01.014

[8] B. Y. H. Lam , A. Williamson , S. Finer , et al., “MC3R Links Nutritional State to Childhood Growth and the Timing of Puberty,” Nature 599, no. 7885 (2021): 436–441. 34732894 10.1038/s41586-021-04088-9 PMC8819628

[9] M. A. Sanchez‐Garrido , V. Serrano‐Lopez , F. Ruiz‐Pino , et al., “Superior Metabolic Improvement of Polycystic Ovary Syndrome Traits After GLP1‐Based Multi‐Agonist Therapy,” Nature Communications 15, no. 1 (2024): 8498. 10.1038/s41467-024-52898-y PMC11445520 39353946

[10] B. Finan , B. Yang , N. Ottaway , et al., “Targeted Estrogen Delivery Reverses the Metabolic Syndrome,” Nature Medicine 18, no. 12 (2012): 1847–1856. 10.1038/nm.3009 PMC3757949 23142820

[11] J. Argente , K. Clément , J. Duis , et al., “Hyperphagia in Rare Melanocortin‐4 Receptor Pathway Diseases: Therapeutic Options and Assessing Treatment Response,” Reviews in Endocrine & Metabolic Disorders 26, no. 6 (2025): 917–935. 40560452 10.1007/s11154-025-09984-3 PMC12602643

[12] S. B. Heymsfield , K. Clément , B. Dubern , et al., “Defining Hyperphagia for Improved Diagnosis and Management of MC4R Pathway–Associated Disease: A Roundtable Summary,” Current Obesity Reports 14, no. 1 (2025): 13. 39856371 10.1007/s13679-024-00601-z PMC11762201

[13] C. Yuan , D. Spiegelman , E. B. Rimm , et al., “Relative Validity of Nutrient Intakes Assessed by Questionnaire, 24‐Hour Recalls, and Diet Records as Compared With Urinary Recovery and Plasma Concentration Biomarkers: Findings for Women,” American Journal of Epidemiology 187, no. 5 (2018): 1051–1063. 29036411 10.1093/aje/kwx328 PMC5928456

[14] L. Matesevac , C. J. Vrana‐Diaz , J. E. Bohonowych , L. Schwartz , and T. V. Strong , “Analysis of Hyperphagia Questionnaire for Clinical Trials (HQ‐CT) Scores in Typically Developing Individuals and Those With Prader‐Willi Syndrome,” Scientific Reports 13, no. 1 (2013): 20573. 10.1038/s41598-023-48024-5 PMC10667498 37996659

[15] S. Zorn , J. von Schnurbein , M. Schirmer , S. Brandt , and M. Wabitsch , “Measuring Hyperphagia in Patients With Monogenic and Syndromic Obesity,” Appetite 178 (2022): 106161. 35809703 10.1016/j.appet.2022.106161

[16] L. I. Arhire , O. Nita , A. D. Popa , et al., “Validation of the Dutch Eating Behavior Questionnaire in a Romanian Adult Population,” Nutrients 13, no. 11 (2021): 3890. 34836141 10.3390/nu13113890 PMC8619088

[17] I. S. Farooqi , G. Matarese , G. M. Lord , et al., “Beneficial Effects of Leptin on Obesity, T Cell Hyporesponsiveness, and Neuroendocrine/Metabolic Dysfunction of Human Congenital Leptin Deficiency,” Journal of Clinical Investigation 110, no. 8 (2002): 1093–1103. 12393845 10.1172/JCI15693 PMC150795

[18] H. Krude , H. Biebermann , W. Luck , R. Horn , G. Brabant , and A. Grüters , “Severe Early‐Onset Obesity, Adrenal Insufficiency and Red Hair Pigmentation Caused by POMC Mutations in Humans,” Nature Genetics 19, no. 2 (1998): 155–157. 9620771 10.1038/509

[19] I. S. Farooqi , J. M. Keogh , G. S. H. Yeo , E. J. Lank , T. Cheetham , and S. O'Rahilly , “Clinical Spectrum of Obesity and Mutations in the Melanocortin 4 Receptor Gene,” New England Journal of Medicine 348, no. 12 (2003): 1085–1095. 12646665 10.1056/NEJMoa022050

[20] S. Farooqi , “Obesity and Thinness: Insights From Genetics,” Philosophical Transactions of the Royal Society of London. Series B, Biological Sciences 378, no. 1888 (2023): 20220205. 37661743 10.1098/rstb.2022.0205 PMC10475868

[21] A. A. van der Klaauw , J. M. Keogh , E. Henning , et al., “Divergent Effects of Central Melanocortin Signaling on Fat and Sucrose Preference in Humans,” Nature Communications 7 (2016): 13055. 10.1038/ncomms13055 PMC5059464 27701398

[22] G. Trivellin , A. F. Daly , F. R. Faucz , et al., “Gigantism and Acromegaly due to Xq26 Microduplications and *GPR101* Mutation,” New England Journal of Medicine 371, no. 25 (2014): 2363–2374. 25470569 10.1056/NEJMoa1408028 PMC4291174

[23] L. Vong , C. Ye , Z. Yang , B. Choi , S. Chua, Jr. , and B. B. Lowell , “Leptin Action on GABAergic Neurons Prevents Obesity and Reduces Inhibitory Tone to POMC Neurons,” Neuron 71, no. 1 (2011): 142–154. 21745644 10.1016/j.neuron.2011.05.028 PMC3134797

[24] L. Garrett , M. Irmler , A. Baljuls , et al., “GPR101 Loss Promotes Insulin Resistance and Diet‐Induced Obesity Risk,” Neuroscience Applied 2 (2023): 101126. 40655975 10.1016/j.nsa.2023.101126 PMC12244024

[25] A. Flores , L. S. Argetsinger , L. K. J. Stadler , et al., “Crucial Role of the SH2B1 PH Domain for the Control of Energy Balance,” Diabetes 68, no. 11 (2019): 2049–2062. 31439647 10.2337/db19-0608 PMC6804625

[26] Y. Yang , A. A. van der Klaauw , L. Zhu , et al., “Steroid Receptor Coactivator‐1 Modulates the Function of Pomc Neurons and Energy Homeostasis,” Nature Communications 10, no. 1 (2019): 1718. 10.1038/s41467-019-08737-6 PMC6461669 30979869

[27] G. Marenne , A. E. Hendricks , A. Perdikari , et al., “Exome Sequencing Identifies Genes and Gene Sets Contributing to Severe Childhood Obesity, Linking PHIP Variants to Repressed POMC Transcription,” Cell Metabolism 31, no. 6 (2020): 1107–1119.e12. 32492392 10.1016/j.cmet.2020.05.007 PMC7267775

[28] A. Bernard , I. O. Naharros , X. Yue , et al., “MRAP2 Regulates Energy Homeostasis by Promoting Primary Cilia Localization of MC4R,” JCI Insight 8, no. 2 (2023): e155900. 36692018 10.1172/jci.insight.155900 PMC9977312

[29] J. E. Siljee , Y. Wang , A. A. Bernard , et al., “Subcellular Localization of MC4R With ADCY3 at Neuronal Primary Cilia Underlies a Common Pathway for Genetic Predisposition to Obesity,” Nature Genetics 50, no. 2 (2018): 180–185. 29311635 10.1038/s41588-017-0020-9 PMC5805646

[30] E. Mendes de Oliveira , J. M. Keogh , F. Talbot , et al., “Obesity‐Associated GNAS Mutations and the Melanocortin Pathway,” New England Journal of Medicine 385 (2021): 1581–1592. 34614324 10.1056/NEJMoa2103329

[31] S. Croizier and S. Bouret , “Molecular Control of the Development of Hypothalamic Neurons Involved in Metabolic Regulation,” Journal of Chemical Neuroanatomy 123 (2022): 102117. 35680104 10.1016/j.jchemneu.2022.102117

[32] C. Quarta , A. Fisette , Y. Xu , et al., “Functional Identity of Hypothalamic Melanocortin Neurons Depends on Tbx3,” Nature Metabolism 1, no. 2 (2019): 222–235. 10.1038/s42255-018-0028-1 PMC8291379 32694784

[33] A. A. van der Klaauw , S. Crozier , E. Mendes de Oliveira , et al., “Human Semaphorin 3 Variants Link Melanocortin Circuit Development and Energy Balance,” Cell 176, no. 4 (2019): 729–742.e18. 30661757 10.1016/j.cell.2018.12.009 PMC6370916

[34] K. M. Brewer , K. K. Brewer , N. C. Richardson , and N. F. Berbari , “Neuronal Cilia in Energy Homeostasis,” Frontiers in Cell and Developmental Biology 10 (2022): 1082141. 36568981 10.3389/fcell.2022.1082141 PMC9773564

[35] K. Clément , E. van den Akker , J. Argente , et al., “Efficacy and Safety of Setmelanotide, an MC4R Agonist, in Individuals With Severe Obesity due to LEPR or POMC Deficiency: Single‐Arm, Open‐Label, Multicentre, Phase 3 Trials,” Lancet Diabetes and Endocrinology 8, no. 12 (2020): 960–970. 33137293 10.1016/S2213-8587(20)30364-8

[36] M. Haqq , W. K. Chung , H. Dollfus , et al., “Efficacy and Safety of Setmelanotide, a Melanocortin‐4 Receptor Agonist, in Patients With Bardet‐Biedl Syndrome and Alström Syndrome: A Multicentre, Randomised, Double‐Blind, Placebo‐Controlled, Phase 3 Trial With an Open‐Label Period,” Lancet Diabetes & Endocrinology 10, no. 12 (2022): 859–868. 36356613 10.1016/S2213-8587(22)00277-7 PMC9847480

[37] J. Argente , C. F. Verge , U. Okorie , et al., “Setmelanotide in Patients Aged 2‐5 Years With Rare MC4R Pathway‐Associated Obesity (VENTURE): A 1 Year, Open‐Label, Multicenter, Phase 3 Trial,” Lancet Diabetes and Endocrinology 13, no. 1 (2025): 29–37. 39549719 10.1016/S2213-8587(24)00273-0

[38] Rhythm Pharmaceuticals, Inc , “A 2‐Stage (Open‐Label Followed by Randomized Double‐Blind, Placebo‐Controlled Stage), Phase 2 Trial of Setmelanotide in Patients With Specific Gene Variants in the Melanocortin‐4 Receptor Pathway,” ClinicalTrials.gov identifier: NCT04963231, accessed March 30, 2025, https://clinicaltrials.gov/study/NCT04963231.

[39] G. Ortiz , S. Ten , W. Herring , et al., “DAYBREAK Trial: Setmelanotide Versus Placebo in Patients With Melanocortin‐4 Receptor Pathway Variants,” Poster presented at ObesityWeek, November 3–6, 2024, accessed July 23, 2025, https://hcp.rhythmtx.com/wp‐content/uploads/sites/2/2024/11/Ortiz_TOS‐2024_Poster‐Daybreak‐Trial‐Part‐2.pdf.

[40] G. Á. Martos‐Moreno , B. Guijo , M. Tena‐Sempere , J. A. Chowen , and J. Argente , “A New Era in Childhood Obesity,” Trends in Endocrinology and Metabolism (2026): S1043‐2760(26)00067‐6, 10.1016/j.tem.2026.03.006. 41927456

[41] I. S. Farooqi , T. Wangensteen , S. Collins , et al., “Clinical and Molecular Genetic Spectrum of Congenital Deficiency of the Leptin Receptor,” New England Journal of Medicine 356, no. 3 (2007): 237–247. 17229951 10.1056/NEJMoa063988 PMC2670197

[42] L. Kleinendorst , O. Abawi , B. van der Voorn , et al., “Identifying Underlying Medical Causes of Pediatric Obesity: Results of a Systematic Diagnostic Approach in a Pediatric Obesity Center,” PLoS One 15, no. 5 (2020): e0232990. 32384097 10.1371/journal.pone.0232990 PMC7209105

[43] S. Courbage , C. Poitou , J. Le Beyec‐Le Bihan , et al., “Implication of Heterozygous Variants in Genes of the Leptin‐Melanocortin Pathway in Severe Obesity,” Journal of Clinical Endocrinology and Metabolism 106, no. 10 (2021): 2991–3006. 34097736 10.1210/clinem/dgab404

[44] S. Zorn , J. C. de Groot , S. Brandt‐Heunemann , et al., “Early Childhood Height, Weight, and BMI Development in Children With Monogenic Obesity: A European Multicentre, Retrospective, Observational Study,” Lancet Child & Adolescent Health 9, no. 5 (2025): 297–305. 40246357 10.1016/S2352-4642(25)00065-3

[45] H. Dollfus , M. R. Lilien , P. Maffei , et al., “Bardet‐Biedl Syndrome Improved Diagnosis Criteria and Management: Inter European Reference Networks Consensus Statement and Recommendations,” European Journal of Human Genetics 32, no. 11 (2024): 1347–1360. 39085583 10.1038/s41431-024-01634-7 PMC11576898

[46] E. Forsythe and P. L. Beales , “Bardet‐Biedl Syndrome,” European Journal of Human Genetics 21, no. 1 (2013): 8–13. 22713813 10.1038/ejhg.2012.115 PMC3522196

[47] M. J. Gurka , C. L. Ice , S. S. Sun , and M. D. DeBoer , “A Confirmatory Factor Analysis of the Metabolic Syndrome in Adolescents: An Examination of Sex and Racial/Ethnic Differences,” Cardiovascular Diabetology 11 (2012): 128. 23062212 10.1186/1475-2840-11-128 PMC3489601

[48] M. J. Gurka , S. L. Filipp , S. K. Musani , M. Sims , and M. D. DeBoer , “Use of BMI as the Marker of Adiposity in a Metabolic Syndrome Severity Score: Derivation and Validation in Predicting Long‐Term Disease Outcomes,” Metabolism 83 (2018): 68–74. 29410278 10.1016/j.metabol.2018.01.015 PMC5960618

[49] M. Haqq , C. Poitou , W. K. Chung , et al., “Impact of Setmelanotide on Metabolic Syndrome Risk in Patients With Bardet‐Biedl Syndrome,” Journal of Clinical Endocrinology and Metabolism 110, no. 10 (2025): e3271–e3282. 39919037 10.1210/clinem/dgaf079 PMC12448622

[50] R. J. F. Loos and G. S. H. Yeo , “The Genetics of Obesity: From Discovery to Biology,” Nature Reviews. Genetics 23, no. 2 (2022): 120–133. 10.1038/s41576-021-00414-z PMC8459824 34556834

[51] S. Richards , N. Aziz , S. Bale , et al., “Standards and Guidelines for the Interpretation of Sequence Variants: A Joint Consensus Recommendation of the American College of Medical Genetics and Genomics and the Association for Molecular Pathology,” Genetics in Medicine 17, no. 5 (2015): 405–424. 25741868 10.1038/gim.2015.30 PMC4544753

[52] F. Mifsud , S. Chalopin , E. Guillon , et al., “Real‐World Efficacy and Safety of Setmelanotide in Adults With Monogenic or Syndromic Obesity: A Prospective Cohort Study,” Obesity (Silver Spring) 33, no. 10 (2025): 1865–1872. 40897637 10.1002/oby.70002

[53] T. Hühne , I. M. Polichronidou , I. Finkelberg , et al., “Impact of the Melanocortin‐4 Receptor (MC4R) Agonist Setmelanotide, on Metabolic Dysfunction‐Associated Steatotic Liver Disease (MASLD) and Kidney Function in Bardet‐Biedl Syndrome,” Journal of Clinical Endocrinology and Metabolism 111, no. 3 (2026): 721–733. 40903014 10.1210/clinem/dgaf483

[54] M. Cetiner , I. Finkelberg , F. Schiepek , L. Pape , R. Hirtz , and A. K. Büscher , “Ultrasound Evaluation of Kidney and Liver Involvement in Bardet–Biedl Syndrome,” Orphanet Journal of Rare Diseases 19, no. 1 (2024): 425. 39533427 10.1186/s13023-024-03400-w PMC11556208

[55] I. Finkelberg , I. M. Polichronidou , T. Hühne , et al., “Patient and Caregiver Experiences With a Patient‐Support Program for Setmelanotide Treatment of Patients With Bardet‐Biedl Syndrome,” Orphanet Journal of Rare Diseases 20, no. 1 (2025): 290. 40484968 10.1186/s13023-025-03835-9 PMC12147271

[56] IMCIVREE (setmelanotide) Summary of Product Characteristics (Rhythm Pharmaceuticals, Inc, 2021), accessed June 3, 2026, https://www.ema.europa.eu/en/documents/product‐information/imcivree‐epar‐product‐information_en.pdf.

[57] H. L. Müller , M. Tauber , E. A. Lawson , et al., “Hypothalamic Syndrome,” Nature Reviews Disease Primers 8, no. 1 (2022): 24. 10.1038/s41572-022-00351-z 35449162

[58] M. J. Abuzzahab , C. L. Roth , and A. H. Shoemaker , “Hypothalamic Obesity: Prologue and Promise,” Hormone Research in Pædiatrics 91, no. 2 (2019): 128–136. 10.1159/000496564 30884480

[59] P. Dimitri , “Treatment of Acquired Hypothalamic Obesity: Now and the Future,” Frontiers in Endocrinology (Lausanne) 13 (2022): 846880. 10.3389/fendo.2022.846880 PMC9019363 35464063

[60] H. M. van Santen , “The Central Control of Energy Metabolism: Hypothalamic Obesity Is Not One Disease,” Hormone Research in Pædiatrics 99, no. 2 (2026): 175–184. 10.1159/000543544 PMC13012768 39819748

[61] J. van Schaik , S. Pillen , R. R. L. van Litsenburg , et al., “The Importance of Specialized Sleep Investigations in Children With a Suprasellar Tumor,” Pituitary 23, no. 6 (2020): 613–621. 32691357 10.1007/s11102-020-01065-9 PMC7585563

[62] H. M. van Santen , J. van Schaik , I. M. A. A. van Roessel , J. Beckhaus , S. Boekhoff , and H. L. Müller , “Diagnostic Criteria for the Hypothalamic Syndrome in Childhood,” European Journal of Endocrinology 188, no. 2 (2023): Ivad009. 10.1093/ejendo/lvad009 36737045

[63] J. van Schaik , A. Y. N. Schouten‐van Meeteren , E. Vos‐Kerkhof , et al., “Treatment and Outcome of the Dutch Childhood Craniopharyngioma Cohort Study: First Results After Centralization of Care,” Neuro‐Oncology 25, no. 12 (2023): 2250–2261. 37381692 10.1093/neuonc/noad112 PMC10708930

[64] I. M. A. A. van Roessel , M. Van Den Brink , J. Dekker , B. G. Ruitenburg‐van Essen , W. J. E. Tissing , and H. M. van Santen , “Feasibility, Safety, and Efficacy of Dietary or Lifestyle Interventions for Hypothalamic Obesity: A Systematic Review,” Clinical Nutrition 43, no. 8 (2024): 1798–1811. 38955055 10.1016/j.clnu.2024.05.028

[65] S. C. Hulsmann , E. W. Hoving , B. Bakker , et al., “BMI Trajectories in the First 3 Months After Childhood Craniopharyngioma Resection: A Plea for Early Management of BMI Changes,” Endocrine Connections 14, no. 4 (2025): e240533. 39952232 10.1530/EC-24-0533 PMC11896649

[66] L. van Iersel , K. E. Brokke , R. A. H. Adan , L. C. M. Bulthuis , E. L. T. van den Akker , and H. M. van Santen , “Pathophysiology and Individualized Treatment of Hypothalamic Obesity Following Craniopharyngioma and Other Suprasellar Tumors: A Systematic Review,” Endocrine Reviews 40, no. 1 (2019): 193–235. 30247642 10.1210/er.2018-00017

[67] C. L. Roth , N. J. Doelman‐Oldenburger , and H. M. van Santen , “Current and Future Pharmacological Interventions for Acquired Hypothalamic Obesity,” Drugs 86, no. 4 (2026): 411–427. 41678022 10.1007/s40265-025-02280-z

[68] Rhythm Pharmaceuticals, Inc , “Rhythm Pharmaceuticals Announces Pivotal Phase 3 TRANSCEND Trial Meets Primary Endpoint with −19.8% Placebo‐adjusted BMI Reduction in Patients (*N*=120) with Acquired Hypothalamic Obesity,” 2025, https://ir.rhythmtx.com/news‐releases/news‐release‐details/rhythm‐pharmaceuticals‐announces‐pivotal‐phase‐3‐transcend‐trial.

[69] S. Phillips , H. M. van Santen , J. K. Hamilton , et al., “Efficacy and Safety of Setmelanotide in Acquired Hypothalamic Obesity: Results from a Double‐Blind, Multicenter, Placebo‐Controlled, Randomized Phase 3 Trial,” Presented at the ENDO Annual Meeting, July 12–15, 2025, Abstract OR11.

[70] H. M. van Santen and H. L. Müller , “Management of Acquired Hypothalamic Dysfunction and the Hypothalamic Syndrome; It Is More Than Obesity,” Endocrine Reviews 46, no. 6 (2025): 891–907. 40746184 10.1210/endrev/bnaf025 PMC12640713

[71] J. Witte , B. Surmann , M. Batram , et al., “Hypothalamic Obesity: Epidemiology in Rare Sellar/Suprasellar Tumors—A German Claims Database Analysis,” Journal of Neuroendocrinology 36, no. 12 (2024): e13439. 39191454 10.1111/jne.13439 PMC11646665

[72] H. L. Müller , J. Witte , B. Surmann , et al., “Treatment of Patients With Tumor/Treatment‐Related Hypothalamic Obesity in the First Two Years Following Surgical Treatment or Radiotherapy,” Scientific Reports 15, no. 1 (2025): 2118. 39814823 10.1038/s41598-025-85262-1 PMC11736136

[73] J. Witte , N. Touchot , B. Surmann , et al., “Economics of Hypothalamic Obesity in Patients With Craniopharyngioma and Other Rare Sellar/Suprasellar Tumors,” European Journal of Health Economics 26, no. 9 (2025): 1557–1567. 10.1007/s10198-025-01786-3 PMC12618323 40343652

[74] J. Witte , N. Touchot , M. Batram , J. Diekmannshemke , M. Flume , and H. L. Müller , “Epidemiology of Acquired Hypothalamic Obesity Following Traumatic Brain Injury and Nonspecific Hypothalamic Microinjury: A Nationwide German Claims Data Analysis,” Journal of Neuroendocrinology 38, no. 1 (2026): e70108. 41236025 10.1111/jne.70108 PMC12799323

[75] H. L. Müller , U. K. Bartels , C. Denzer , et al., “Expert Meeting Report: Epidemiology and Management of Acquired Hypothalamic Obesity,” Frontiers in Endocrinology (Lausanne) 17 (2026): 1798619. 10.3389/fendo.2026.1798619 PMC13124477 42064767

[76] T. Tanaka , K. Takeda , H. Takagi , et al., “An Estimated Epidemiology of Adult Acquired Hypothalamic Obesity in Japan—A Database Analysis,” Presented at: 98th Annual Congress of the Japan Endocrine Society; June 5–7, 2025, Abstract O1‐6‐1.

[77] P. Faucher , S. Chalopin , F. Albarel , et al., “Real‐World Setmelanotide Changes in BMI in French Patients With Acquired Hypothalamic Obesity,” Endocrine Abstracts 110 (2025): JOINT2287.

[78] C. L. Roth , C. Scimia , A. H. Shoemaker , et al., “Setmelanotide for the Treatment of Acquired Hypothalamic Obesity: A Phase 2, Open‐Label, Multicentre Trial,” Lancet Diabetes and Endocrinology 12, no. 6 (2024): 380–389. 38697184 10.1016/S2213-8587(24)00087-1

[79] C. L. Roth and S. E. McCormack , “Acquired Hypothalamic Obesity: A Clinical Overview and Update,” Diabetes, Obesity and Metabolism 26, no. S2 (2024): 34–45. 10.1111/dom.15530 PMC13372222 38450938

[80] P. Kühnen , K. Clément , S. Wiegand , et al., “Proopiomelanocortin Deficiency Treated With a Melanocortin‐4 Receptor Agonist,” New England Journal of Medicine 375, no. 3 (2016): 240–246. 27468060 10.1056/NEJMoa1512693

[81] K. Clément , H. Biebermann , I. S. Farooqi , et al., “MC4R Agonism Promotes Durable Weight Loss in Patients With Leptin Receptor Deficiency,” Nature Medicine 24, no. 5 (2018): 551–555. 10.1038/s41591-018-0015-9 29736023

[82] Rhythm Pharmaceuticals, Inc , “A Phase 2, Randomized, Double‐Blind, Placebo‐Controlled Pilot Study to Assess the Effects of RM‐493, a Melanocortin 4 Receptor (MC4R) Agonist, in Obese Subjects With Prader‐Willi Syndrome (PWS) on Safety, Weight Reduction, and Food‐Related Behaviors,” ClinicalTrials.gov identifier: NCT02311673, accessed March 30, 2025, https://clinicaltrials.gov/study/NCT02311673.

[83] J. M. Bischof , L. H. Van Der Ploeg , W. F. Colmers , and R. Wevrick , “Magel2‐Null Mice Are Hyper‐Responsive to Setmelanotide, a Melanocortin 4 Receptor Agonist,” British Journal of Pharmacology 173, no. 17 (2016): 2614–2621. 27339818 10.1111/bph.13540 PMC4978157

[84] Rhythm Pharmaceuticals, Inc , “A Phase 2 Open‐Label Study of Setmelanotide in Patients With Prader‐Willi Syndrome,” ClinicalTrials.gov identifier: NCT06772597, accessed July 27, 2025, https://clinicaltrials.gov/study/NCT06772597.

[85] F. Juurians , G. F. Kerkhof , and A. C. S. Hokken‐Koelega , “The Spectrum of the Prader‐Willi‐Like Pheno‐ and Genotype: A Review of the Literature,” Endocrine Reviews 43, no. 1 (2022): 1–18. 34460908 10.1210/endrev/bnab026

